# Coencapsulation of doxorubicin and curcumin in liposomes modified with folic acid for reversal of drug resistance in glioma

**DOI:** 10.3389/fonc.2026.1856255

**Published:** 2026-06-17

**Authors:** Zhan Wang, Ze Zhao, Yang Yang, Yi Zhao

**Affiliations:** 1 Department of Pharmacy, the First Affiliated Hospital of Zhengzhou University, Zhengzhou, China; 2 Department of Orthopedics, the First Affiliated Hospital of Henan Polytechnic University (the Second People’s Hospital of Jiaozuo City), Jiaozuo, China; 3 Department of Translational Medicine Center, the First Affiliated Hospital of Zhengzhou University, Zhengzhou, China

**Keywords:** curcumin, doxorubicin, doxorubicin resistance, folic acid, glioma

## Abstract

**Introduction:**

Multidrug resistance (MDR) critically limits doxorubicin (Dox) efficacy in glioma. To overcome this, we constructed a folic acid receptor (FAR)-targeted liposomal system for codelivering Dox and curcumin (Cur).

**Methods:**

Dox/Cur-Lip@FA was prepared using the film hydration-sonication method. The physicochemical properties (size, zeta potential, encapsulation efficiency, and drug release) were characterized. The studies assessed P-gp modulation and cellular uptake in Dox-resistant C6 cells, as well as *in vivo* efficacy in xenograft models.

**Results:**

The FA ligand enabled selective tumor targeting by binding to overexpressed FAR, while Cur enhanced Dox efficacy by downregulating P-gp-mediated drug efflux. Dox/Cur-Lip@FA exhibited a uniform size of 112.5 ± 3.8 nm, a zeta potential of -7.85 ± 0.62 mV, high dual-drug EE% (Dox: 91.32 ± 3.95%; Cur: 90.87 ± 4.21%), and sustained release kinetics. *In vitro*, targeted liposomes achieved 3.56-fold higher cellular uptake in Dox-res-C6 cells compared to non-targeted formulations. In an *in vitro* BBB model, FA modification significantly enhanced transendothelial transport, suggesting potential for brain delivery. *In vivo*, Dox/Cur-Lip@FA induced a 71.19% reduction in tumor volume, significantly outperforming free drugs and non-targeted liposomes. Mechanistic analysis confirmed that Cur effectively suppressed P-gp expression, thereby restoring Dox sensitivity.

**Conclusion:**

This FA-functionalized codelivery system combines active targeting with MDR reversal, showing improved cellular uptake *in vitro* and antitumor efficacy in subcutaneous xenografts, with potential for BBB penetration as suggested by *in vitro* models.

## 1 Introduction

Glioma, the most common primary malignant brain tumor, poses severe therapeutic challenges because of its high invasiveness and inherent resistance to chemotherapy (1). Doxorubicin (Dox), a broad-spectrum anthracycline antibiotic, is widely used for glioma treatment because it intercalates into DNA and inhibits topoisomerase II. However, over 60% of patients develop Dox resistance during treatment, which drastically limits its clinical efficacy (2). The primary mechanism of Dox resistance in glioma involves P-glycoprotein (P-gp), an ATP-binding cassette (ABC) transporter that mediates ATP-driven efflux of Dox and other hydrophobic drugs, reducing intracellular drug accumulation (3). Additionally, the blood–brain barrier (BBB) and glioma tumor microenvironment further hinder drug delivery to tumor sites, exacerbating therapeutic failure (4).

Current strategies to reverse Dox resistance in glioma have notable limitations: systemic administration of P-gp inhibitors causes severe off-target toxicity, whereas nontargeted drug delivery systems fail to penetrate the BBB and achieve effective drug concentrations in glioma tissues (5, 6). MDR in glioma is driven by multiple interconnected mechanisms, including enhanced drug efflux via ABC transporters (P-gp, MRPs, and BCRP), reduced drug uptake, altered apoptotic signaling pathways, and metabolic reprogramming (7). Among these factors, P-gp is the most well-documented and dominant factor, making it a key target for MDR reversal (8).

In recent years, nanoliposomal systems have emerged as a promising solution to address the challenges of delivering Dox to gliomas. Liposomal encapsulation improves Dox solubility, reduces systemic toxicity (especially cardiotoxicity), and extends the circulation time. Moreover, liposomes can passively accumulate in tumor tissues via the enhanced permeability and retention (EPR) effect and can be modified with targeting ligands to cross the BBB and bind to glioma-specific receptors (9, 10). Curcumin (Cur), a natural polyphenol derived from Curcuma longa, has attracted attention for its dual role in glioma therapy: it exhibits inherent antiglioma activity by inhibiting cell proliferation and inducing apoptosis and acts as a potent chemosensitizer by downregulating P-gp expression and inhibiting its ATPase activity (11, 12). While liposomal Dox and Cur have been independently studied for glioma treatment, the codelivery of both agents in a single targeted liposomal platform—combining active targeting and MDR reversal—remains underdeveloped.

The folic acid receptor (FAR) is a glycosylphosphatidylinositol-anchored protein that is overexpressed on many glioma cells and tumor neovasculature but shows limited expression in normal tissues, making it a suitable target for active drug delivery (13, 14). C6 glioma cells express functional folate receptors, and several studies have used FR−targeted nanocarriers for drug delivery to C6 glioma models *in vitro* and *in vivo* (15–19). For example, folic acid−conjugated cellulose nanocrystals were shown to enter C6 cells via FR−mediated endocytosis, with free folic acid inhibiting a significant portion of cellular uptake (20). These data support the feasibility of using FA−functionalized liposomes to actively target FAR−expressing C6 glioma cells. Conjugating folic acid to liposomes enables receptor-mediated endocytosis, promoting tumor-selective drug internalization and accumulation while reducing off-target uptake (21).

Several studies have already used FA-targeted nanocarriers for glioblastoma. FA-modified liposomes and polymeric nanoparticles, for example, can improve drug delivery to FAR-overexpressing glioma cells *in vitro* and *in vivo*, leading to better outcomes than non-targeted formulations (16, 18, 22). However, most of these systems deliver only a single drug and do not address the multidrug resistance (MDR) mechanisms that limit chemotherapy effectiveness. Only a few FA-targeted platforms include a P-gp modulator to reverse doxorubicin resistance (23). Even in combination strategies, the full potential of linking active targeting with MDR reversal through a chemosensitizer remains largely unexplored. Our study differs from these earlier efforts by using a new FA-functionalized liposome that co-delivers doxorubicin and curcumin. Here, curcumin acts both as an antitumor agent and as a P-gp downregulator. This design simultaneously achieves FAR-mediated targeting and reverses P-gp-driven drug efflux.

**Figure 1:**
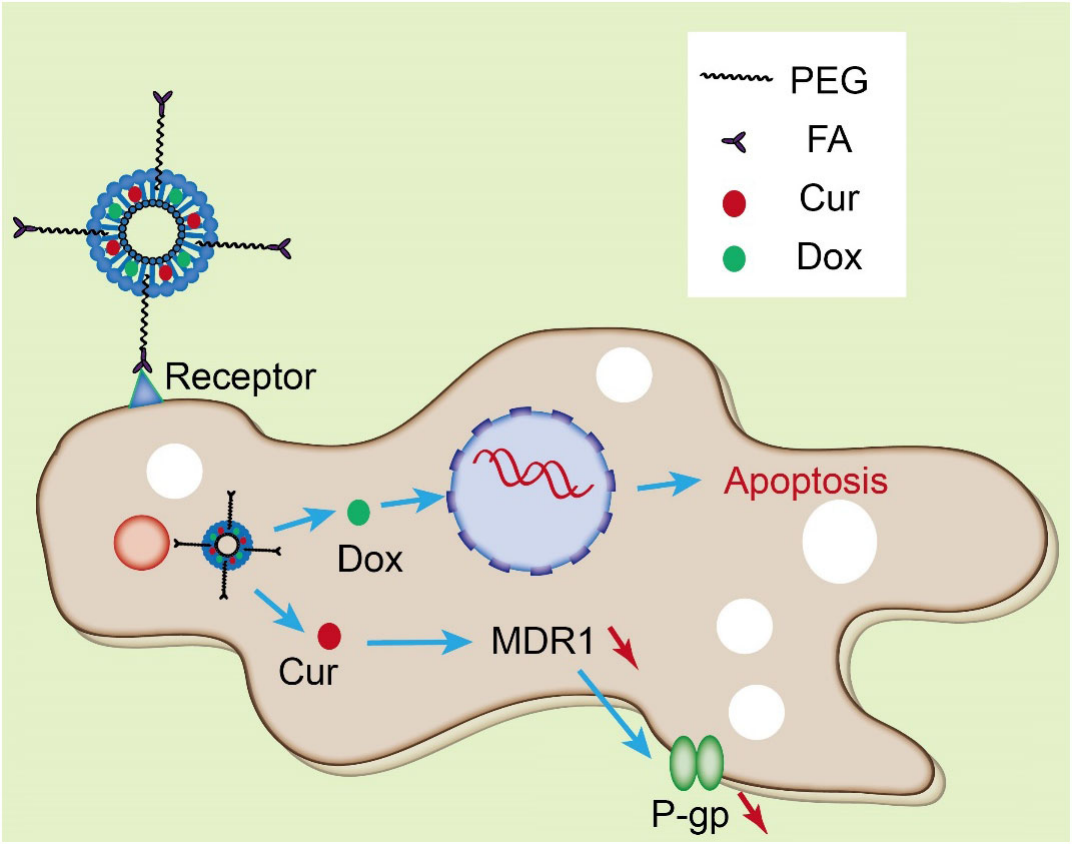
Illustration of the ability of Dox/Cur-Lip@FA to target glioma.

To address Dox resistance in glioma, which is driven by P-gp-mediated drug efflux and poor drug delivery, this study developed a novel FA-functionalized liposomal system that coloads Dox and Cur (Dox/Cur-Lip@FA) for targeted delivery to FAR-overexpressing Dox-res-C6 cells (**Figure 1**). The novelty of this work lies in its three-pronged mechanism: (1) FA-mediated active targeting to potentially enhance glioma-specific drug uptake and, based on *in vitro* models, possibly facilitate BBB transport; (2) Cur-mediated P-gp suppression to reduce Dox efflux; and (3) synchronized codelivery of Dox and Cur via a single nanocarrier to maximize synergistic efficacy. This integrated strategy addresses key barriers to glioma therapy and was validated through *in vitro* and *in vivo* experiments. We demonstrated that Dox/Cur-Lip@FA restored Dox sensitivity in resistant glioma cells, suppressed P-gp expression, and exhibited potent *in vivo* antitumor activity, providing a new paradigm for targeted MDR reversal in glioma.

## 2 Methods

### 2.1 Materials

Doxorubicin hydrochloride (Dox), curcumin (Cur), cholesterol, and soybean phospholipids (SPC) were purchased from Sangon Biotech (Shanghai) Co., Ltd. (China). 1,2-dioleoyl-sn-glycero-3-phosphoethanolamine-N-(carboxyfluorescein) (CFPE) was obtained from Avanti Polar Lipids (Alabaster, AL, USA). High-performance liquid chromatography (HPLC) analyses were performed on a Waters Alliance e2695 system (Waters Corp., Milford, MA, USA) equipped with an Agilent ZORBAX Eclipse Plus C18 column (200 × 4.6 mm, 5 μm). The mobile phase for Dox detection was acetonitrile/0.1% trifluoroacetic acid (40:60, v/v) at a flow rate of 1.0 mL/min (detection wavelength: 480 nm); for Cur detection, the mobile phase was methanol/water (80:20, v/v) at 1.0 mL/min (detection wavelength: 425 nm).

Dox-resistant C6 cells (Dox-res-C6) were induced by stepwise exposure of parental C6 cells to increasing DOX concentrations (0.01–0.5 μM) over 6 months, and stable resistance was confirmed by the MTT assay. The cells were cultured in RPMI-1640 medium supplemented with 10% fetal bovine serum (FBS) and 1% penicillin–streptomycin and maintained in a 5% CO_2_ incubator at 37 °C.

All animal experiments were approved by the Animal Ethics Committee of the Second People’s Hospital of Jiaozuo City (approval no.: KY2024-07-090; date: 31 July 2024). Male BALB/c nude mice (6–8 weeks old, 18–22 g) were purchased from Charles River Laboratories (Beijing, China) and housed under specific pathogen-free (SPF) conditions (temperature: 22 ± 2 °C, humidity: 50 ± 5%, 12 h light/dark cycle) with free access to food and water.

### 2.2 Chemistry

#### 2.2.1 Synthesis of cholesteryl tosylate (2)

The cholesteryl tosylate (2) was prepared according to our previous report (24). Cholesterol 1 (1.00 g, 2.59 mmol) was introduced into anhydrous pyridine (10 mL) in a three-neck flask under nitrogen. A solution of p-toluenesulfonyl chloride (0.74 g, 3.89 mmol) in pyridine (10 mL), prepared separately, was added dropwise over 30 minutes via a pressure-equalizing addition funnel while the temperature was maintained at 50 °C. The mixture was mechanically stirred for 12 hours. After the reaction was complete, the volatiles were removed under vacuum. The semisolid residue was removed in ethyl acetate (50 mL) and washed consecutively with 1 M HCl (3 × 15 mL) and saturated NaCl (3 × 15 mL). The organic phase was dried over anhydrous MgSO_4_, filtered through a medium-porosity filter, and concentrated to yield compound 2 (1.09 g, 92%). This material was used directly in further steps. HPLC indicated a purity of 93%. ESI-MS (m/z): calcd 540.36, observed 540.81. Elemental analysis: Calcd (%) C, 75.51; H, 9.69; S, 5.93; Found: C, 75.72; H, 9.60; S, 5.97.

#### 2.2.2 Synthesis of PEG2000-cholesteryl (3)

A mixture of compound 2 (1.00 g, 1.85 mmol) and PEG2000 (5.55 g, 2.78 mmol) in anhydrous 1,4-dioxane (50 mL) was refluxed with vigorous mechanical stirring for 8 hours under argon. After cooling to room temperature, the solvent was removed under reduced pressure. The residue was dissolved in dichloromethane (100 mL), and the organic layer was washed with saturated sodium chloride solution (3 × 15 mL), dried over anhydrous sodium sulfate, and concentrated via rotary evaporation. Purification by silica gel chromatography afforded compound 3 as a colorless viscous oil (1.22 g, 52%). HPLC analysis revealed a purity of 91%. ESI-MS (m/z): calcd 2367.53; found 2366.22. Elemental analysis: calcd C 59.32, H 9.62; found C 59.17, H 9.73.

#### 2.2.3 Synthesis of FA-PEG2000-cholesteryl (4)

To a solution containing folic acid (0.5 g, 1.13 mmol) and compound 3 (1.34 g, 0.57 mmol) in dimethyl sulfoxide (DMSO, 30 mL), ethyldimethylaminopropyl carbodiimide (EDC, 85 mg, 0.57 mmol) and N-hydroxy succinimide (NHS, 80 mg, 0.68 mmol) were introduced. The resulting mixture was stirred at room temperature for 12 hours. After filtration to remove the byproduct, the filtrate was treated with diethyl ether to precipitate a yellow solid. Purification via flash chromatography afforded FA-PEG2000-Cholesteryl (4) (0.65 g, 41%) as a colorless viscous oil. HPLC analysis revealed a purity of 91%. ESI-MS (m/z): calcd 2790.66; found 2791.83. Elemental analysis: calcd C, 58.50; H, 8.77; N, 3.51; found C, 58.34; H, 8.59; N, 3.63.

### 2.3 Preparation of doxorubicin and curcumin coloaded liposomes (Dox/Cur-Lip@FA)

Dox/Cur-Lip@FA was prepared via the film hydration–sonication method with slight modifications (24). The formulation components included soybean phospholipids (15 mg), cholesterol (3 mg), FA-PEG2000-Cholesteryl (9 mg), Dox (0.9 mg), and Cur (0.19 mg). All the components were dissolved in a mixture of chloroform and methanol (1:1, 10 mL) in a round-bottom flask. The solvent was evaporated under reduced pressure (37 °C, 0.08 MPa) via a rotary evaporator to form a uniform thin film, which was further dried under vacuum for 5 hours to remove residual solvent. The film was hydrated with 12 mL of distilled water (preheated to 37 °C) under gentle stirring for 40 minutes to form crude liposomes. The crude liposomes were sonicated using a probe sonicator (90 W, 100 s, with a 2 s on/3 s off cycle) to reduce the particle size. They were then centrifuged at 12,000 rpm for 15 minutes to remove unentrapped drugs and large aggregates.

Nontargeted liposomes (Dox/Cur-Lip) were prepared via the same method but with FA-PEG2000-Cholesteryl replaced with PEG2000-Cholesteryl (3). The drug encapsulation efficiency (EE%) was determined via high-performance liquid chromatography (HPLC): 0.5 mL of the liposome suspension was lysed with 0.5 mL of methanol and centrifuged at 10,000 rpm for 10 minutes, after which the Dox and Cur concentrations in the supernatant were analyzed. Drug loading capacity (DL%) was defined as the mass ratio of encapsulated drug to total lipids. DL% was calculated using the formula: DL% = (mass of encapsulated drug/total lipid mass) × 100%. EE% was calculated as follows: EE% = (encapsulated drug amount/total drug amount) × 100%. The particle size, polymer dispersity index (PDI), and zeta potential were measured via a Malvern Zetasizer Nano ZS90 (Malvern Instruments, UK) at 25 °C.

### 2.4 *In vitro* drug release from Dox/Cur-Lip@FA

The *in vitro* release kinetics of Dox and Cur from liposomal formulations were assessed via a dialysis membrane technique (25). In brief, 0.5 mL of Dox/Cur-Lip@FA, Dox/Cur-Lip, or the Dox/Cur combination was sealed in a dialysis bag (MWCO: 8,000–14,000 Da) and immersed in 50 mL of release medium (PBS with 0.1% Tween 80) at 37 °C under continuous shaking at 50 rpm. Three different pH conditions were evaluated: pH 7.4 (physiological), pH 6.0 (tumor microenvironment), and pH 5.5 (endosomal/lysosomal). At predetermined intervals (0, 1, 2, 4, 8, 12, 24, and 48 h), 200 μL of the medium was sampled and replaced with an equal volume of fresh buffer. The concentrations of Dox and Cur in the collected samples were quantified via HPLC. The cumulative release percentage was calculated via the following formula: Cumulative release (%) = (Amount of drug released/Total drug loaded) × 100%.

### 2.5 Stability of liposomes in serum *in vitro*

The stability of Dox/Cur-Lip@FA and Dox/Cur-Lip was evaluated in biological media by tracking changes in turbidity. The liposomes were combined with fetal bovine serum (FBS) at a 1:1 volume ratio in PBS (pH 7.4) and shaken (45 rpm) at 37 °C. Samples (200 μL) were taken after 0, 1, 2, 4, 8, 12, 24, and 48 hours and transferred to a 96-well plate, and the transmittance at 750 nm was recorded via a BioTek microplate reader (USA). The transmittance retention percentage was determined as follows: (Transmittance at time t/Initial transmittance) × 100%. A higher retention of transmittance reflects greater liposome stability and reduced aggregation.

### 2.6 Hemolysis assays

The hemocompatibility of the liposomes was assessed via a hemolysis assay using mouse red blood cells (RBCs). Fresh mouse blood (2 mL) anticoagulated with 3.8% sodium citrate (1:9 v/v) was obtained via orbital sinus puncture. The blood was centrifuged at 3,000 rpm for 10 min to isolate RBCs, which were then washed three times with PBS (pH 7.4) and resuspended in PBS to achieve a 2% (v/v) suspension. Different amounts of liposomes (10, 25, 50, 100, 200, and 400 nmoles in 0.4 mL) were combined with 0.1 mL of the 2% RBC suspension and maintained at 37 °C for 2 hours. PBS and 1% Triton X-100 served as the negative and positive controls, respectively. After incubation, the samples were centrifuged at 10,000 rpm for 10 min, and the absorbance of the supernatant was recorded at 540 nm. The percentage of hemolysis was determined via the following formula: Hemolysis% = [(A_sample_ - A_negative_)/(A_positive_ - A_negative_)] × 100%, where A denotes the absorbance value at 540 nm. A hemolysis rate below 10% was deemed indicative of good biocompatibility (17).

### 2.7 Cytotoxicity assay

The cytotoxic effects of various formulations on Dox-resistant C6 cells (Dox-res-C6) and L929 mouse fibroblasts cell were assessed via the MTT assay. The cells were plated in 96-well plates at 5 × 10³ cells per well and cultured overnight. The culture medium was then replaced with fresh medium containing the following formulations: Dox, Dox/Cur (3:1, molar ratio), Dox/Cur-Lip, and Dox/Cur-Lip@FA. The Dox concentration varied from 0.1 to 200 nM (0.1, 0.2, 0.5, 1, 5, 20, 50, 100, 200 nM). For the Dox/Cur combination groups (free or encapsulated), the molar ratio of Dox to Cur was fixed at 3:1, and the concentrations of both drugs were normalized based on the molar amount of Dox to ensure a fair comparison of biological activity across all formulations. Following 24 hours of treatment, 20 μL of MTT solution was added to each well, and the plates were incubated for another 4 hours. After the supernatant was removed, 150 μL of DMSO was added to solubilize the formazan crystals. The absorbance was recorded at 570 nm via a microplate reader. Cell viability was determined via the following formula: Viability % = (A_treated_/A_control_) × 100%, where A_treated_ is the absorbance of drug-treated wells and A_control_ is that of untreated wells. IC_50_ values were determined by nonlinear regression using a four−parameter logistic model (log[inhibitor] vs. normalized response).

### 2.8 Calcein/PI staining

Dox-res-C6 cells in the logarithmic growth phase were plated in 6-well plates at a density of 1 × 10^5^ cells per well and maintained for 24 hours. The culture medium was then replaced with serum-free medium containing the following formulations: Dox, Dox/Cur (3:1, molar ratio), Dox/Cur-Lip, and Dox/Cur-Lip@FA, each at a Dox concentration of 5 nM. Following 24 hours of exposure, the medium was aspirated, and the cells were subjected to three washes with assay buffer (from the Calcein/PI Staining Kit, Beyotime, China). A working staining solution comprising 2 μmol/L Calcein-AM and 4.5 μmol/L propidium iodide (PI) was applied, and the cells were incubated in the dark at 37 °C for 30 minutes. After three additional washes with PBS, images were acquired via an inverted fluorescence microscope (Olympus IX73, Japan). Viable cells were labeled green by Calcein-AM, whereas dead cells were stained red by PI.

### 2.9 P-gp level detection

Dox-res-C6 cells were plated in 6-well plates at a density of 4 × 10^5^ cells per well and maintained for 24 hours. The culture medium was then replaced with serum-free medium containing different formulations at a Dox concentration of 5 nM; the control group received drug-free, serum-free medium. Serum−free conditions were applied to exclude potential modulatory effects of serum−derived factors on P−gp expression, allowing isolation of the direct effect of curcumin. Following 24 hours of treatment, the cells were collected, washed twice with PBS, and subjected to three freeze–thaw cycles (-80 °C to 37 °C) for lysis. The lysate was centrifuged at 2,000 rpm for 20 minutes, and the supernatant was retrieved. The P−gp level was quantified using a commercial mouse P−gp ELISA kit (DLdevelop, China) based on a double−antibody sandwich method. Briefly, P−gp in cell lysates was captured by the pre−coated anti−P−gp antibody, then detected with a biotinylated secondary antibody and HRP−conjugated streptavidin. After adding TMB (Tetramethylbenzidine) substrate and stop solution, the absorbance was measured at 450 nm via a microplate reader, and relative P-gp expression was calculated as follows: relative P-gp expression% = (A_sample_/A_blank_) × 100%, where A_sample_ represents the absorbance of the sample and A_blank_ corresponds to the blank control.

### 2.10 Cellular uptake study

Dox-res-C6 cells were seeded into 12-well plates at a density of 5 × 10^5^ cells per well and allowed to adhere for 24 hours until they reached approximately 80% confluence. The culture medium was then replaced with serum-free medium containing either CFPE-Lip or CFPE-Lip@FA, with a final CFPE concentration of 2 μg/mL. Serum−free conditions were used to prevent protein corona formation and free−folate competition, ensuring an accurate evaluation of FA−mediated targeting. Following 4 hours of incubation at 37 °C, the cells were rinsed three times with ice-cold PBS, detached via trypsin, and collected via centrifugation at 1,500 rpm for 5 minutes. The resulting cell pellets were resuspended in 0.5 mL of PBS and analyzed via a BD FACS Celestra flow cytometer (USA). The fluorescence was measured at an excitation wavelength of 495 nm and an emission wavelength of 515 nm. The cellular uptake efficiency was assessed on the basis of the mean fluorescence intensity, with all experiments conducted in triplicate.

To visually assess the cellular internalization of the liposomes, fluorescence microscopy was performed. After incubation with CFPE-labeled liposomes under the same conditions as described above, the cells were washed with PBS and fixed with 4% paraformaldehyde. Nuclei were stained with DAPI, and the cellular uptake was visualized via a fluorescence microscope. Images were captured and processed to evaluate the distribution and intensity of CFPE fluorescence within the cells.

### 2.11 *In vitro* permeability study of the BBB model

The *in vitro* BBB model was established via Millicell hanging cell culture inserts. Briefly, bEnd.3 cells were plated on 6-well cell culture inserts at a density of 2×10^6^ cells per well and cultured for 7 days at 37 °C. The transendothelial electrical resistance (TEER) of bEnd.3 monolayers was assessed via a Millicell ERS system (Millipore, USA), and only monolayers exhibiting TEER values above 200 Ω were employed as valid BBB models for subsequent experiments. Moreover, C6 cells were seeded in separate 6-well plates. The inserts containing bEnd.3 monolayers were then transferred to the plate with C6 cells and cocultured for an additional 24 h. Afterward, CFPE-Lip or CFPE-Lip@FA was introduced into the donor chamber and incubated for 4 h. Both bEnd.3 and C6 cells were rinsed three times with cold PBS, detached using trypsin, and resuspended in PBS. Finally, the intracellular fluorescence density was measured to evaluate the drug delivery efficiency.

### 2.12 Antitumor activity *in vivo*

To establish Dox-res-C6 xenograft models, nude mice were anesthetized with isoflurane (induction: 4% in oxygen, maintenance: 1.5–2% in oxygen) via a vaporizer and then subcutaneously inoculated in the right flank with 1 × 10^7^ Dox-res-C6 cells suspended in 100 μL of a 1:1 (v/v) PBS/Matrigel mixture. When the tumors reached approximately 50 mm³, the mice were randomly assigned to five groups (n = 6 per group): (1) Saline control; (2) Dox; (3) Dox/Cur; (4) Dox/Cur-Lip; and (5) Dox/Cur-Lip@FA. All the treatments were delivered via tail vein injection every three days over four cycles. The dosage of Dox was maintained at 3 mg/kg per injection, while Cur was administered at 1 mg/kg (molar ratio 3:1). The doses were normalized based on the molar amount of Dox to ensure consistent comparison of antitumor activity across all treatment groups.

Tumor dimensions and body weights were recorded every two days. The tumor volume was determined via the following formula: (Length × Width²)/2. On day 21, mice were euthanized by gradual carbon dioxide (CO_2_) inhalation (20% chamber volume displacement per minute) followed by cervical dislocation to ensure death. The absence of respiration and heartbeat was confirmed before tumor collection.

### 2.13 Statistical analysis

Statistical analyses used GraphPad Prism 8.0. Data are mean ± SD (n = 3 *in vitro*, n = 6 *in vivo*). Normality and variance homogeneity were assessed by Shapiro–Wilk and Levene’s tests, respectively. One−way ANOVA with Tukey’s *post hoc* test was applied for multiple comparisons. Exact p−values are reported in the text and figure legends; p < 0.05 was considered significant. *In vivo* sample size (n = 6/group) was determined by power analysis (G*Power 3.1, effect size 1.2, α = 0.05, β = 0.20, 80% power), which gave a minimum of 5 mice per group; we added one extra to account for potential dropout.

## 3 Results

### 3.1 Chemistry

**Figure 2:**
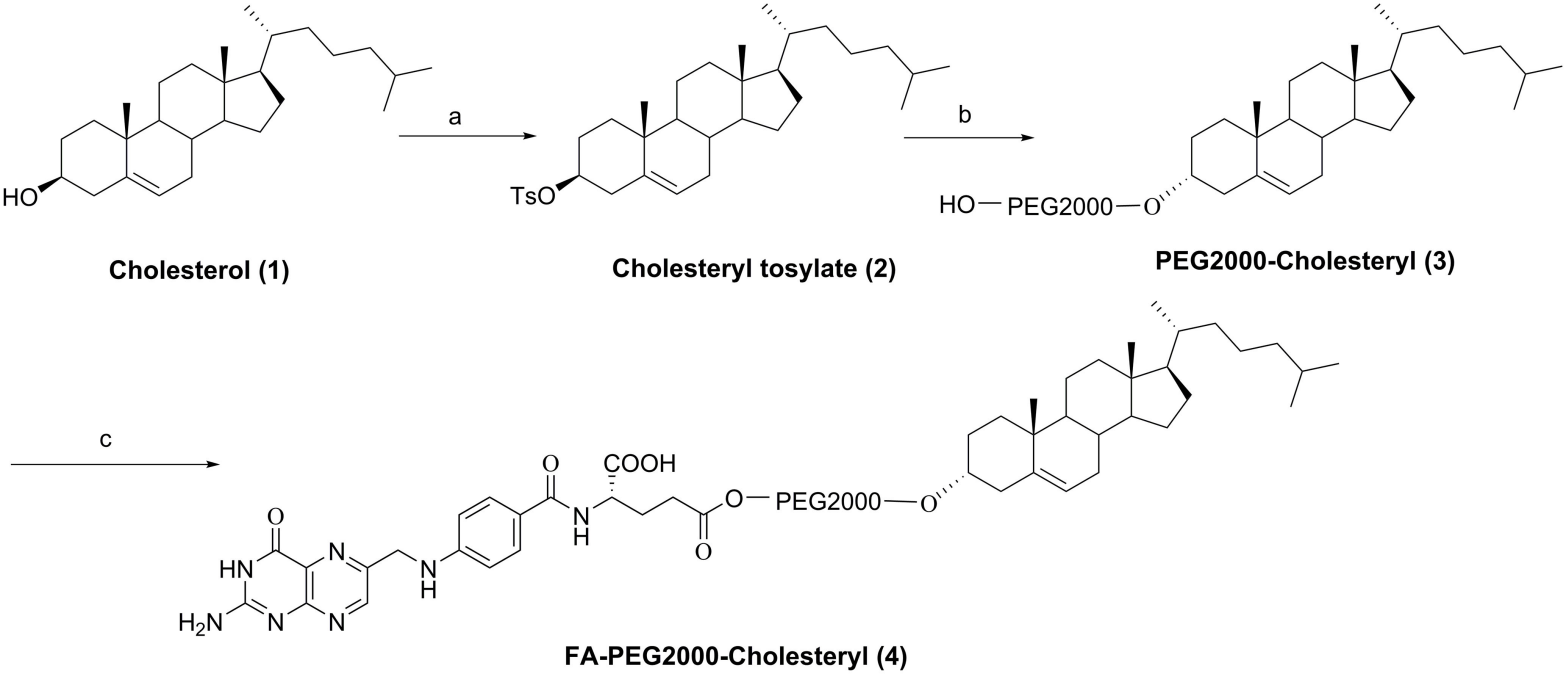
Preparation of the liposome ligand FA-PEG2000-Cholesteryl. Reagents and conditions: **(a)** p-toluenesulfonyl chloride, pyridine, 50 °C, 12 h. **(b)** PEG2000, 1,4-dioxane, reflux, 8 h. **(c)** Folic acid, EDC, NHS, and DMSO, r.t., 12 h.

The synthetic route for the folate-modified liposome ligand FA-PEG2000-Cholesteryl is illustrated in **Figure 2**. The key transformations involved (1) esterification of cholesterol via p-toluenesulfonyl chloride to afford cholesteryl tosylate, which introduces a suitable leaving group for subsequent nucleophilic substitution; (2) esterification of 2 with PEG2000 to form PEG2000-Cholesteryl, which incorporates a hydrophilic spacer to increase the solubility and prolong the circulation time; and (3) conjugation of 3 with folic acid (FA) via carbodiimide-mediated coupling to produce the target ligand FA-PEG2000-Cholesteryl.

The carboxylic acid groups of FA served as the reactive sites for esterification. The reaction employed EDC/NHS activation to form an active ester intermediate, which subsequently reacted with the hydroxyl terminus of PEG under mild conditions to form a stable ester bond. This approach preserved the integrin-targeting capability of FA. The purities of all the intermediates and the final product exceeded 90%, and structural confirmation was achieved through ESI–MS and elemental analysis.

### 3.2 Preparation and characterization of liposomes

The physicochemical characteristics of the Dox/Cur-Lip and Dox/Cur-Lip@FA are presented in **Table 1**. Both liposomal preparations had consistent particle sizes of approximately 110 nm, falling within the ideal size range (100–200 nm) for enhanced tumor targeting via the EPR effect and penetration across the blood–brain barrier (26). The PDI values were approximately 0.2, which meets the accepted criterion for monodisperse nanocarriers (PDI < 0.3) (26), indicating satisfactory size uniformity for *in vivo* applications. Formulations with PDI > 0.3 were excluded from further experiments. The zeta potential of Dox/Cur-Lip@FA (–7.85 ± 0.62 mV) was slightly more negative than that of Dox/Cur-Lip (–2.21 ± 0.38 mV), likely due to the presence of carboxyl groups from FA. A negative zeta potential contributes to minimized liposome aggregation and reduced protein opsonization, thereby prolonging the circulation time *in vivo* (25).

**Table 1:** Physicochemical properties of Dox/Cur-Lip and Dox/Cur-Lip@FA (n=3, mean ± SD).

Group	Size (nm)	EE (%)		PDI	DL (%)		Zeta potential (mV)
		Dox	Cur		Dox	Cur	
Dox/Cur-Lip	105.7 ± 5.2	90.45 ± 3.82	91.03 ± 4.05	0.198 ± 0.012	3.01 ± 0.12	0.64 ± 0.02	-2.21 ± 0.38
Dox/Cur-Lip@FA	112.5 ± 3.8	91.32 ± 3.95	90.87 ± 4.21	0.196 ± 0.009	3.04 ± 0.13	0.64 ± 0.03	-7.85 ± 0.62

**Figure 3:**
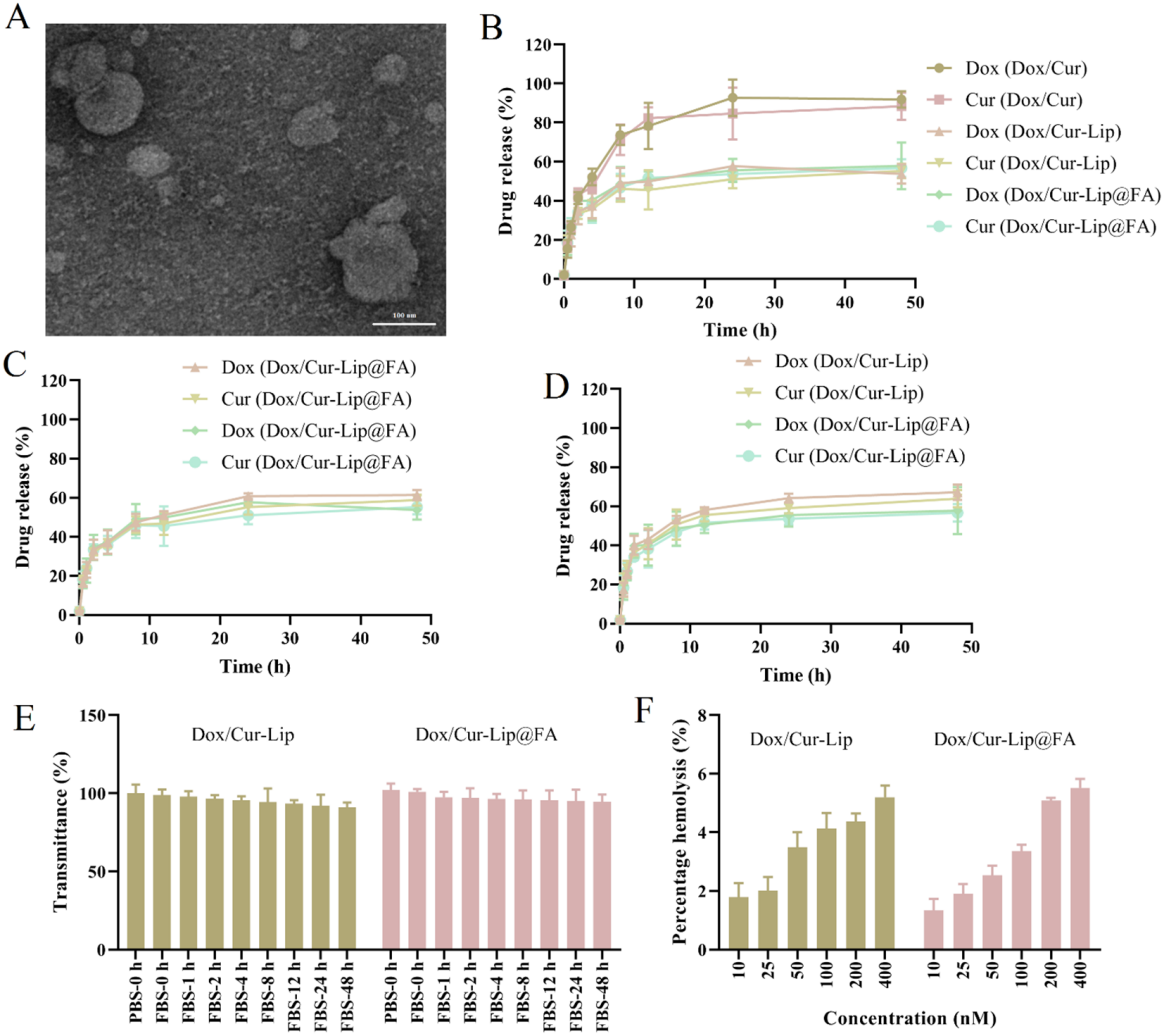
*In vitro* characterization of liposomes. **(A)** TEM image of Dox/Cur-Lip@FA. **(B)** *In vitro* release profiles of Dox and Cur from different formulations (pH 7.4). **(C)** *In vitro* release profiles of Dox and Cur from different formulations (pH 6.0). **(D)** *In vitro* release profiles of Dox and Cur from different formulations (pH 5.5). **(E)** Transmittance retention rates of liposomes in 50% FBS. **(F)** Hemolysis rates of liposomes at different lipid concentrations. The data are presented as the means ± SDs (n=3). No statistical comparisons were made in this figure; data are descriptive only.

High encapsulation efficiency was attained for both drugs in each formulation: the Dox: EE% reached 90.45 ± 3.82% for Dox/Cur-Lip and 91.32 ± 3.95% for Dox/Cur-Lip@FA, whereas the Cur: EE% was 91.03 ± 4.05% and 90.87 ± 4.21%, respectively. The DL% of Dox/Cur-Lip@FA were 3.01 ± 0.12% for Dox and 0.64 ± 0.02% for Cur, comparable to those of non−targeted Dox/Cur−Lip (Dox: 3.04 ± 0.13%, Cur: 0.64 ± 0.03%). Although the Cur loading capacity appears modest, curcumin is known to exert potent P−gp downregulation at low doses, thus the formulation maintains a favorable balance between delivery efficiency and pharmacological activity. A spherical morphology and homogeneous size distribution were observed in the TEM images (**Figure 3A**) of Dox/Cur-Lip@FA, which was in agreement with the DLS results.

The *in vitro* drug release profiles are presented in **Figure 3B**. Free Dox and Cur were rapidly released, with cumulative amounts exceeding 78% and 82%, respectively, within 12 hours. In comparison, both liposomal formulations demonstrated sustained release patterns: Dox/Cur-Lip released approximately 53% of the Dox and 55% of the Cur over 48 hours, whereas Dox/Cur-Lip@FA released approximately 57% of the Dox and 56% of the Cur. An initial burst release (0–4 h) was observed, likely resulting from superficially adsorbed drugs, followed by a slower phase governed by diffusion through the lipid bilayer—a hallmark of liposomal delivery systems (13). No notable difference in release profiles was found between the two liposomal groups, suggesting that FA modification did not alter drug release kinetics. We further examined the release of Dox/Cur-Lip and Dox/Cur-Lip@FA at pH 6.0 and pH 5.5. For both formulations, the release patterns at acidic pH remained similar to that at pH 7.4, but the cumulative release was higher. The accelerated release under acidic conditions is likely due to protonation of lipid headgroups and destabilization of the liposomal bilayer, which promotes drug diffusion. This pH−responsive behavior is beneficial for antitumor therapy, as it facilitates drug release after liposome internalization into acidic tumor sites or endosomes, thereby improving intracellular drug bioavailability and therapeutic efficacy.

Serum stability is crucial for *in vivo* applications since liposome aggregation can induce premature drug leakage and enhance clearance by the reticuloendothelial system (RES). As shown in **Figure 3E**, both Dox/Cur-Lip and Dox/Cur-Lip@FA retained transmittance values above 90% over 48 hours in 50% FBS, indicating negligible aggregation and high colloidal stability. This effect is ascribed to the PEG2000 layer, which provides steric hindrance that reduces serum protein adsorption (27).

Hemocompatibility is essential for intravenous delivery. As depicted in **Figure 3F**, the hemolysis rates for both liposomal formulations remained below 10% at lipid concentrations up to 400 nmoles, which is substantially under the 10% safety limit. These findings demonstrate that Dox/Cur-Lip@FA has favorable physicochemical properties, controlled release, serum stability, and blood compatibility, supporting its potential for *in vivo* use.

### 3.3 Cytotoxicity assay

**Figure 4:**
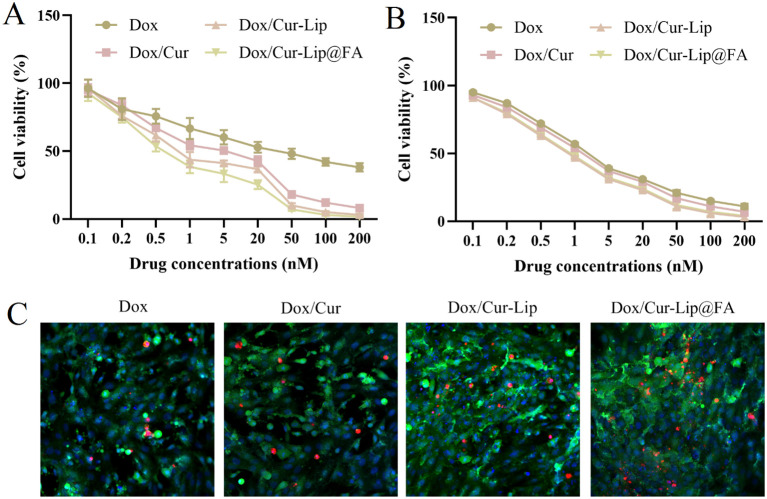
Cytotoxicity and live/dead staining of the liposomal formulations. **(A)** Cytotoxicity of different formulations against Dox-res-C6 cells. **(B)** Cytotoxicity of different formulations against L929 cells. **(C)** Calcein/PI staining images.

The cytotoxicity of different formulations against Dox−resistant C6 cells was evaluated by MTT assay over a Dox concentration range of 0.1–200 nM (**Figure 4A**). Free Dox alone showed limited efficacy, with cell viability remaining above 34% even at the highest concentration (200 nM). The IC_50_ of free Dox was determined to be 66.8 ± 3.2 nM. The free Dox/Cur combination significantly enhanced potency, reducing the IC_50_ to 2.38 ± 0.19 nM. Encapsulation of Dox/Cur into non−targeted liposomes (Dox/Cur−Lip) further lowered the IC_50_ to 0.77 ± 0.08 nM, while the FA−targeted liposomes (Dox/Cur−Lip@FA) achieved the lowest IC_50_ of 0.57 ± 0.06 nM, representing an ~117−fold improvement over free Dox and a ~4.2−fold improvement over the free drug combination. These IC_50_ values show a progressive recovery of Dox sensitivity when combining active targeting with P−gp downregulation (12). Free Dox and free Dox/Cur showed no selectivity, with comparable or even greater cytotoxicity against L929 than against Dox-res-C6 cells. In contrast, both liposomal formulations killed Dox-res-C6 cells more efficiently than L929 cells at clinically relevant concentrations (0.2–1 nM), and Dox/Cur−Lip@FA showed the greatest difference, confirming that FA−targeted codelivery selectively kills resistant glioma cells while sparing normal fibroblasts (**Figure 4B**).

Notably, Dox/Cur-Lip@FA displayed the strongest cytotoxicity. This superior performance is due to FA-driven active targeting: FA binds to folic acid receptors overexpressed on Dox-resistant C6 cells, facilitating receptor-mediated endocytosis and increasing intracellular drug delivery (14). Moreover, coadministration of Cur further inhibited P-gp expression, limiting Dox efflux and augmenting cytotoxic effects. Together, these findings underscore that integrating active targeting with multidrug resistance reversal markedly enhances the efficacy of Dox in resistant glioma cells.

### 3.4 Calcein/PI staining

Calcein/PI staining (**Figure 4C**) corroborated the cytotoxicity outcomes. The cells treated with free Dox presented abundant green fluorescent viable cells and few red fluorescent dead cells, suggesting limited toxicity toward Dox-res-C6 cells. Compared with the free Dox alone group, the Dox/Cur combination group presented a greater proportion of red-stained cells, confirming the role of Cur as a chemosensitizer. Dox/Cur-Lip treatment resulted in a further increase in cytotoxicity, whereas Dox/Cur-Lip@FA produced the most extensive red staining and the lowest number of viable green cells. These visual results demonstrate that Dox/Cur-Lip@FA enables effective codelivery of Dox and Cur to resistant cells, promoting substantial cell death and overcoming multidrug resistance.

### 3.5 P-gp level detection

**Figure 5:**
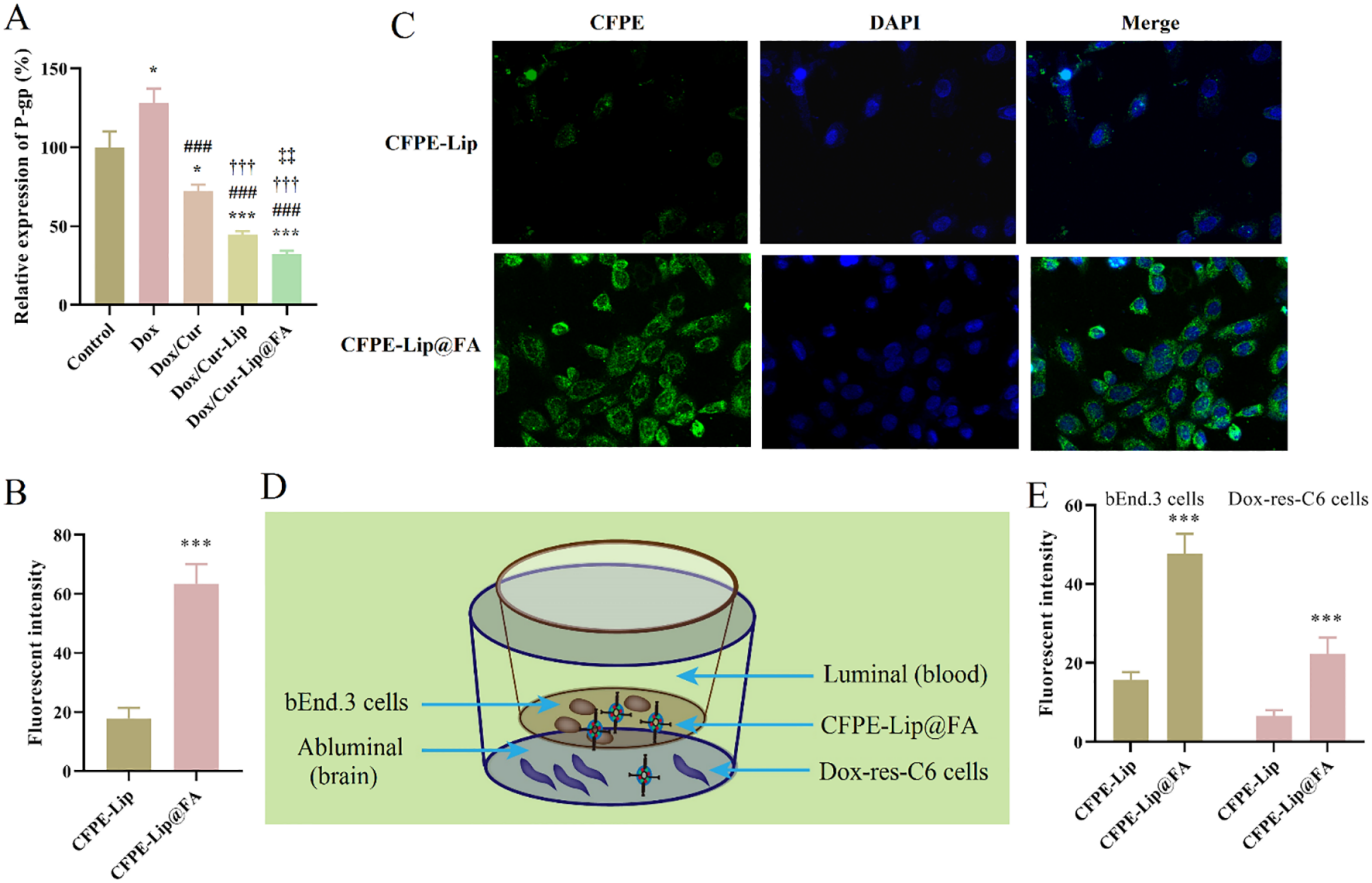
P-gp expression and cellular uptake of liposomal formulations. **(A)** Relative P-gp expression levels. **(B)** Cellular uptake of CFPE-labeled liposomes. **(C)** Qualitative image of cellular uptake. **(D)** *In vitro* BBB model. **(E)** Uptake by bEnd.3 cells and C6 cells in the presence of the BBB model. Data are presented as mean ± SD (n=3). Statistical significance was determined by one-way ANOVA followed by Tukey’s *post hoc* test. In **(A)**: *p < 0.05, ***p < 0.001 vs. Control group; ^###^p < 0.001 vs. Dox group; ^†††^p < 0.001 vs. Dox/Cur group; ^‡‡^p < 0.01 vs. Dox/Cur-Lip group. In **(B)**: ***p < 0.001 vs. CFPE-Lip group. In **(E)**: ***p < 0.001 vs. CFPE-Lip group.

P-gp overexpression is a major driver of Dox resistance in Dox-res-C6 cells. To investigate the reversal of MDR, we evaluated P-gp expression (**Figure 5A**). Compared with the control, Dox significantly increased P-gp levels, likely due to drug-induced upregulation, which promotes efflux (28). In contrast, the free Dox/Cur combination group presented reduced P-gp expression, confirming the inhibitory role of Cur. Liposomal delivery of Dox/Cur further decreased P-gp, owing to improved Cur uptake.

Compared with the free Dox group, the Dox/Cur-Lip@FA group presented the strongest suppression, representing a 74.68% reduction. This enhanced effect stems from two factors: FA-mediated targeting improves Cur delivery, potentiating P-gp downregulation, which in turn reduces Dox efflux, leading to increased intracellular accumulation and cytotoxicity. This self-reinforcing cycle—enhanced Cur delivery, suppressed P-gp, increased Dox retention, and improved efficacy—underscores the system’s ability to overcome MDR. Thus, Cur-loaded liposomes effectively reverse Dox resistance by downregulating P-gp.

### 3.6 Cellular uptake

Cellular internalization efficiency is vital for therapeutic outcomes, particularly in resistant cells with impaired drug uptake. Using CFPE-labeled liposomes, we assessed uptake via flow cytometry (**Figure 5B**). The fluorescence microscopy images (**Figure 5C**) further supported these results, revealing intense punctate fluorescence associated with Lip@FA, which was consistent with enhanced endocytic activity. The CFPE-Lip@FA group presented a fluorescence intensity of 63.42, a 3.56-fold increase over that of CFPE-Lip. This increase was attributed to FA-FAR binding: FA engages receptors on membranes, initiating receptor-mediated endocytosis and accelerating liposome entry (15). In contrast, nontargeted CFPE-Lips internalize via passive diffusion or pinocytosis, resulting in lower efficiency.

### 3.7 *In vitro* permeability study of the BBB model

The integrity of the bEnd.3 monolayers was confirmed by TEER values exceeding 200 Ω, ensuring a reliable BBB model for coculture with C6 cells (**Figure 5D**). Following 4 h of incubation, both CFPE-Lip and CFPE-Lip@FA were internalized by the cells, as detected via intracellular fluorescence. In an *in vitro* BBB model using bEnd.3 monolayers, CFPE-Lip@FA showed higher transendothelial transport compared to non-targeted liposomes. These results suggest a potential benefit of FA modification for BBB crossing (**Figure 5E**). This result underscores the potential of FA-modified liposomes for improving drug delivery to brain tumors.

### 3.8 Antitumor activity *in vivo*

**Figure 6:**
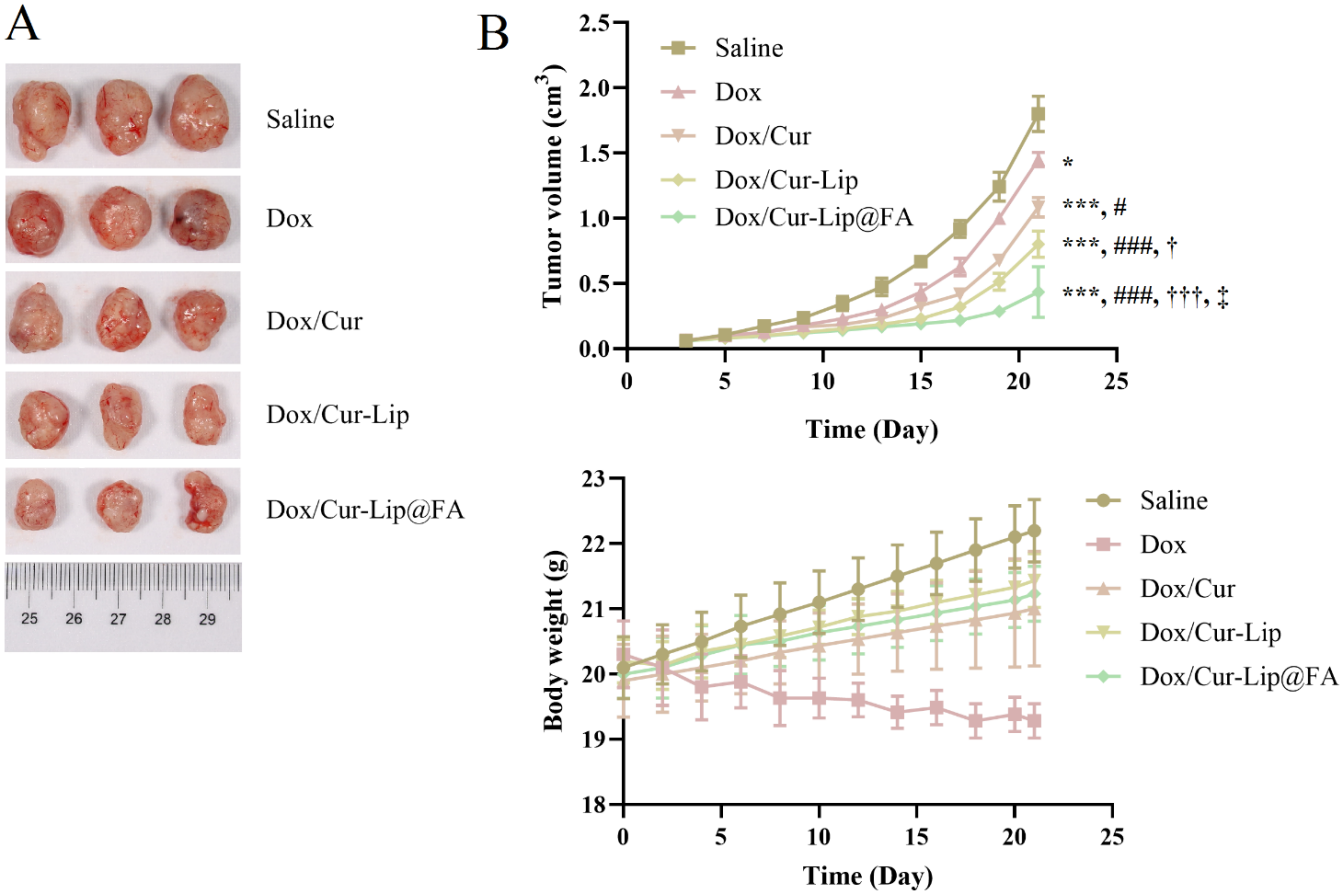
*In vivo* antitumor efficacy of different formulations. **(A)** Representative tumor images on day 21. **(B)** Tumor growth curves and body weight changes over 21 days. Data are presented as mean ± SD (n=6). Statistical significance was determined by one-way ANOVA followed by Tukey’s *post hoc* test. *p < 0.05, ***p < 0.001 vs. Saline group; ^#^p < 0.05, ^###^p < 0.001 vs. Dox group; ^†^p < 0.05, ^†††^p < 0.001 vs. Dox/Cur group; ‡p < 0.05 vs. Dox/Cur-Lip group.

The reversal of tumor multidrug resistance (MDR) *in vivo* is depicted in **Figure 6**. Mice treated with saline showed rapid and continuous tumor growth, consistent with the behavior of untreated aggressive tumor models. The administration of free Dox results in limited antitumor activity owing to P-gp-mediated drug efflux and intrinsic resistance mechanisms. In comparison, the combination of Dox/Cur led to significantly greater suppression of tumor progression than did free Dox alone. These results indicate that Cur functions as an effective chemosensitizer by reducing P-gp expression and suppressing drug efflux, thus partially reversing chemoresistance. Most importantly, the targeted liposomal system Dox/Cur-Lip@FA achieved the highest tumor inhibition rate at 71.19%, markedly outperforming both free drug treatments (**Figure 6A**). This enhanced efficacy originates from the synergy between active tumor targeting via the FA ligand and optimized codelivery of Dox and Cur. The formulation effectively counteracts MDR through a dual approach: promoting tumor-selective drug uptake and blocking efflux pathways, leading to substantially elevated intracellular drug concentrations and cytotoxic effects. As shown in **Figure 6B**, body weight changes were monitored over 21 days. The Saline group showed a gradual increase in body weight (from 20.1 ± 0.5 g to 22.2 ± 0.5 g), consistent with normal growth. The Dox group showed a slight decrease (from 20.3 ± 0.5 g to 19.3 ± 0.3 g), while the Dox/Cur, Dox/Cur-Lip, and Dox/Cur-Lip@FA groups maintained stable body weights throughout the treatment period. No significant differences in body weight were observed between any treatment group and the Saline group at any time point.

## 4 Discussion

In this study, we developed a folic acid-modified liposome co-delivery system (Dox/Cur-Lip@FA) to reverse multidrug resistance in glioma. This system exhibited excellent physicochemical properties: the particle size was approximately 112 nm, the entrapment efficiency of both drugs exceeded 90%, and it demonstrated sustained-release behavior. Folic acid modification increased the uptake of liposomes by Dox-res-C6 cells by 3.56 times compared to the non-targeted group, indicating that folic acid receptor-mediated endocytosis played a crucial role. Meanwhile, curcumin significantly downregulated the expression of P-gp by 74.68%, thereby restoring the sensitivity of drug-resistant cells to doxorubicin. In the subcutaneous tumor model, the tumor growth inhibition rate of Dox/Cur-Lip@FA reached 71.19%, and its therapeutic effect was significantly better than that of free drugs and non-targeted liposomes.

This enhanced therapeutic effect can be understood from two synergistic mechanisms. On the one hand, folate-mediated active targeting facilitates the selective delivery of the drug to glioma cells with high expression of folate receptors, which is consistent with the literature reports on the expression of functional folate receptors in C6 cells and the utilization of this target by FA-modified nanocarriers (15–19). Notably, the 3.56-fold increase in uptake observed by us has certain advantages over some earlier FA-targeting systems (20). On the other hand, curcumin, as a chemosensitizer, downregulates P-gp, which is in line with the known effect of Cur in inhibiting P-gp expression (11, 12). Dox/Cur-Lip@FA reduces P-gp expression by 74.68%, an effect that is not inferior to and even better than some other Cur-based delivery systems (28), possibly due to the enhanced intracellular delivery efficiency of Cur by FA targeting.

Unlike previous FA-targeting platforms that only deliver single drugs (16, 18, 22) or lack built-in drug resistance regulation functions (23), this system simultaneously achieves active targeting and MDR reversal within a single carrier. From the perspective of *in vivo* anti-tumor efficacy, the tumor suppression rate of Dox/Cur-Lip@FA reached 71.19%, significantly higher than the free Dox/Cur combination and non-targeted liposomes. This improvement is particularly prominent in the research of FA-targeting nanocarriers for glioma. For example, although FA-modified exosomes enhanced the efficacy of temozolomide, they did not solve the problem of P-gp-mediated drug resistance (22); FA-modified gold nanoclusters, as a radiosensitizer, also lacked chemosensitizing effects (18). Therefore, this system combines targeting with drug resistance reversal, filling a relatively critical technological gap.

Of course, this study also has several limitations. Firstly, the *in vivo* experiments used a subcutaneous ectopic tumor model, which cannot fully simulate the microenvironment of intracranial gliomas and the actual situation of the blood-brain barrier. Although the results of our *in vitro* BBB model suggest that FA modification is helpful for transendothelial transport, it is still necessary to further verify the brain distribution and therapeutic effect of the drug in the *in situ* tumor model. Secondly, we mainly focused on P-gp, but glioma multidrug resistance may also involve other ABC transporters such as MRP1, BCRP, etc. (7, 8), and it is not clear whether curcumin has similar inhibitory effects on them. Nevertheless, published evidence suggests that curcumin can also inhibit MRP1 and BCRP, and broader transporter profiling could be pursued in future studies. Thirdly, the long-term safety of the FA-PEG2000-cholesterol conjugate needs to be evaluated through chronic toxicity experiments. Fourthly, the scalability of the film hydration-ultrasound preparation process is also a problem that needs to be considered for future clinical translation. Future work should focus on verifying the *in situ* glioma model, conducting a more comprehensive analysis of the transporter inhibition spectrum, conducting long-term toxicity evaluation, and exploring whether this strategy is also applicable to other solid tumors with high P-gp expression.

## 5 Conclusion

In summary, we developed an FA-targeted liposome co-loaded with doxorubicin and curcumin (Dox/Cur-Lip@FA) to overcome multidrug resistance in glioma. The system achieved high encapsulation efficiency (>90%), sustained release, FA-mediated cellular uptake (3.56-fold increase), and P−gp suppression via curcumin, leading to a 71.19% tumor growth reduction *in vivo*—better than free drugs or non-targeted liposomes. Unlike previous FA carriers that lack a built−in resistance modulator, our platform directly links active targeting with chemosensitization. Limitations include lack of brain distribution data in orthotopic models, no assessment of other ABC transporters (e.g., MRP1, BCRP), and unknown long−term safety of the FA-PEG ligand. Future work should use orthotopic models to evaluate BBB crossing, study broader transporter inhibition, and conduct chronic toxicity tests. For clinical translation, the scalability of the preparation still needs to be addressed. Whether this strategy works for other P−gp−overexpressing cancers requires separate investigation.

## Data Availability

The original contributions presented in the study are included in the article/supplementary material. Further inquiries can be directed to the corresponding author.
